# Correlation of serum chloride concentrations with components of the renin‐angiotensin‐aldosterone system in a dog with congestive heart failure

**DOI:** 10.1111/jvim.17238

**Published:** 2024-10-31

**Authors:** Cailey Banken, Autumn N. Harris, Rachel Conway, Eduardo J. Benjamin, Robin Shoemaker, Darcy Adin

**Affiliations:** ^1^ Department of Small Animal Clinical Sciences, College of Veterinary Medicine University of Florida Gainesville Florida USA; ^2^ Department of Pediatrics, College of Medicine University of Kentucky Lexington Kentucky USA; ^3^ Present address: North Carolina State University, College of Veterinary Medicine Raleigh North Carolina USA

**Keywords:** diuretics, electrolytes, hypochloremia, RAAS

## Abstract

A 7‐year‐old male castrated Cavalier King Charles Spaniel was hospitalized for 12 days for treatment of severe congestive heart failure secondary to myxomatous mitral valve disease. During that time, 6 serum samples from different days were analyzed for serum biochemical and renin‐angiotensin‐aldosterone system components. Serum chloride concentrations (ranging from 71.6 to 103.1 mmol/L) were inversely related to angiotensin I concentrations, aldosterone concentrations, a surrogate marker of renin activity, and a surrogate marker of adrenal responsiveness to angiotensin II. In light of recent studies showing that hypochloremia is associated with advanced heart failure in dogs and is associated with poor outcomes in people, the information from the dog in this report supports exploration of RAAS dysregulation as an underlying mechanism.

**Abbreviations**
AA2: aldosterone to angiotensin II ratio as an indicator of adrenal responsiveness to angiotensin II
ACE‐S: angiotensin‐converting enzyme activity surrogate calculated as angiotensin II divided by angiotensin I
CHF: congestive heart failure
Cl^−^
: chloride
PRA‐S: plasma renin activity surrogate calculated as the sum of angiotensin I and angiotensin II
RAAS: renin‐angiotensin aldosterone system

## 1 INTRODUCTION

Recent studies in people and dogs demonstrate that serum chloride concentrations ([Cl^−^]) are altered in congestive heart failure (CHF), worsening disease progression and outcomes in people.^1^, ^2^, ^3^, ^4^, ^5^, ^6^, ^7^ Hypochloremia is promoted by loop diuretics, which waste chloride through urinary excretion, and by drug or disease‐induced activation of the renin‐angiotensin‐aldosterone system (RAAS), leading to nonosmotic release of antidiuretic hormone and electrolyte dilution.^8^, ^9^, ^10^ Sensing of adequate chloride in the distal tubular fluid by the macula densa cells of the kidney occurs by binding the chloride molecule to with‐no‐lysine kinase K, preventing phosphorylation.^11^, ^12^ However, inadequate chloride allows these kinases to be phosphorylated, which activates RAAS.^12^ Therefore, there is interest in determining if restoring serum [Cl^−^] to normal will improve patient outcomes by quieting the RAAS in CHF. We present successive serum [Cl^−^], angiotensin I, angiotensin II, and aldosterone measurements in a dog with varying degrees of hypochloremia during in‐hospital treatment for CHF.

## 2 CASE PRESENTATION

A 7‐year‐old male castrated Cavalier King Charles Spaniel weighing 8.2 kg was presented to the University of Florida, Small Animal Hospital after being treated by its primary care veterinarian for acute left‐sided CHF secondary to severe degenerative mitral valve disease. Before the presentation, over a 24‐hour period, the dog received a cumulative dose of 12 mg/kg furosemide (Salix, Merck Animal Health, Rahway, NJ) IV, torsemide (Demadex, Camber Pharm., Piscataway, NJ; 0.15 mg/kg PO twice), pimobendan (Vetmedin, Boehringer Ingelheim, Duluth, GA; 0.29 mg/kg PO q8h), and oxygen therapy. Because the dog remained clinically affected by unresolved CHF at presentation, it was placed in an oxygen cage for continued diuretic therapy after admission to our hospital. The dog received an additional cumulative dose of 10 mg/kg furosemide (Salix, Merck Animal Health, Rahway, NJ) administered as a continuous IV infusion over 17 hours in addition to pimobendan (Vetmedin, Boehringer Ingelheim, Duluth, GA; 0.3 mg/kg PO q8h), which resolved radiographic and clinical signs of CHF. The dog was then transitioned to PO furosemide (Salix, Merck Animal Health, Rahway, NJ; 2.4 mg/kg) on the 2nd day of hospitalization. Echocardiography showed severe degenerative mitral valve disease with severe mitral regurgitation, severe left ventricular enlargement, and severe left atrial enlargement. Two small thrombi were noted on the left atrium wall, so the dog was prescribed clopidogrel (Plavix, Aurobindo Pharma, East Windsor, NJ; 2.3 mg/kg PO q24h).

The dog developed concurrent suspected pancreatitis, which prompted the initiation of supportive therapy, including nasogastric feeding, ondansetron (Zofran, GlaxoSmithKlein, Zebulon, NC), maropitant citrate (Cerenia, Zoetis Inc, Parsippany‐Troy Hills, NJ), capromorelin (Entyce, Elanco, Greenfield, IN), buprenorphine (Buprenex, Par Pharmaceuticals, Chestnut Ridge, NY), methadone (Mylan, Morgantown, WV), pantoprazole (Protonix, Aspiro Pharma, Telanga, IN), mirtazapine (Remeron, Aurobindo, East Windsor, NJ), and metoclopramide (Reglan, Hospira, Lake Forest, IL). Because of progressive electrolyte abnormalities (Table 1), potassium chloride supplementation (Fresenius Labs, Lake Zurich, IL; 0.3 mEq/kg PO q12h) and tolvaptan (Samsca, Phar Pharmaceuticals, Chestnut Ridge, NY; 1.8 mg/kg PO q12h) were started on Day 4, the PO furosemide (Salix, Merck Animal Health, Rahway, NJ) was reduced to 1.2 mg/kg q12h, and amlodipine (Norvsac, Phar Pharmaceuticals, Chestnut Ridge, NY) 0.1 mg/kg q24h was started on Day 5 of hospitalization. Tolvaptan (Samsca, Phar Pharmaceuticals, Chestnut Ridge, NY) was discontinued after 2 doses because of the development of hematuria which then resolved. The bloodwork values reflected advanced CHF and loop diuretic administration, with varying degrees of azotemia, hyponatremia, hypokalemia, hyperphosphatemia, and alkalosis at most time points. The dog was variably hypochloremic at all time points ([Cl^−^] ranged from 71.6 to 103.1 mmol/L). The dog improved with supportive therapy and was discharged to home care after 12 days of hospitalization. The dog successfully underwent open heart surgery for mitral valve repair 7 months after presentation.

**TABLE 1:** Body weight, serum biochemical variables, and renin‐angiotensin‐aldosterone system components were measured during hospitalization for congestive heart failure.

Day of hospitalization	1	5	6	9	10	12
Body weight (kg)	8.2	7.5	7.8	7.7	7.7	7.7
Glucose (78‐124 mg/dL)	105	138	132	102	96	76
Albumin (2.6‐3.9 g/dL)	3.21	3.09	3.11	2.83	2.72	2.94
BUN (7‐27 mg/dL)	32	58	82	74	55	30
Creatinine (0.6‐1.5 mg/dL)	0.93	1.70	2.14	1.48	1.58	1.23
Sodium (142‐151 mEq/L)	139.4	124.5	128.8	141.1	140.6	145.2
Potassium (3.8‐5.0 mEq/L)	3.1	3.4	3.5	3.6	3.5	3.8
Chloride (107.9‐117.1)	88.3	71.6	78.0	98.4	98.0	103.1
Corrected chloride	92.6	84.1	88.6	102.0	101.9	103.8
Bicarbonate (16‐24 mEq/L)	33	36	34	26	28	24
Calcium (8.7‐10.4 mg/dL)	9.8	9.9	10.6	9.8	9.6	10.4
Phosphorus (2.2‐4.8 mg/dL)	5.9	5.4	5.9	4.6	3.6	3.4
Anion gap	21.2	20.3	20.3	20.3	18.1	21.9
Calculated osmolality	296.1	277.4	294.2	314.3	306.2	305.3
Angiotensin I, pmol/L	3164.3	4624.1	2038.8	1838.3	1450.2	576.1
Angiotensin II, pmol/L	566.4	580.7	862.9	704.9	728.7	397.2
Aldosterone, pmol/L	1132.5	2758.7	1661.9	1387.3	243.9	188.6
PRA‐S, pmol/L	3730.7	5204.8	2901.7	2543.2	2178.9	973.3
ACE‐S (pmol/L)/(pmol/L)	0.18	0.13	0.42	0.38	0.50	0.69
AA2 (pmol/L)/(pmol/L)	2.00	4.75	1.93	1.97	0.33	0.47

*Note*: Units and reference ranges are provided in the first column.

Abbreviations: AA2, aldosterone to angiotensin II ratio; ACE‐S, angiotensin‐converting enzyme activity surrogate calculated as angiotensin II divided by angiotensin I; BUN, blood urea nitrogen; PRA‐S, renin activity surrogate calculated as the sum of angiotensin I and angiotensin II.

Serum samples collected by peripheral venipuncture on 6 different days during the 12‐day hospitalization period (Days 1, 5, 6, 9, 10, and 12) were analyzed for glucose, albumin, blood urea nitrogen, creatinine, sodium, potassium, chloride, bicarbonate, calcium, phosphorus, and anion gap using an AU480 Chemistry Analyzer (Beckman Coulter, Brea, California) through the Clinical Pathology Laboratory at the University of Florida Small Animal Hospital. Serum osmolality was calculated (calOsm) = 2[sodium] + [glucose/18] + [blood urea nitrogen/2.8] and serum [Cl^−^] was mathematically corrected for the serum sodium concentration using the formula: corrected [Cl^−^] = (midreference range sodium/measured sodium) × measured [Cl^−^]. With client consent, residual serum was used to quantify concentrations of angiotensin I, angiotensin II, and aldosterone at equilibrium, using liquid chromatography/tandem mass spectrometry‐based assay that has been previously described (RAAS Triple‐ATM, Attoquant Diagnostics GmbH, Vienna, Austria), analyzed by a service core laboratory at the University of Kentucky on a TSQ Altis Plus (Thermo Fisher Scientific, Waltham, MA).^13^ Concentrations of individual analytes were used to calculate biomarkers reflecting activity of the RAAS as previously reported.^13^, ^14^ The sum of angiotensin I and angiotensin II was used as a surrogate for plasma renin activity (PRA‐S), a biomarker for overall activity of the RAAS. The angiotensin II to angiotensin I ratio was used as a surrogate for angiotensin‐converting enzyme (ACE) activity (ACE‐S) and the aldosterone to angiotensin II ratio (AA2) was used as an indicator of adrenal responsiveness to angiotensin II. Table 1 shows measured and calculated variables on each of the 6 days. The dog was heterozygous positive for the known ACE polymorphism.^15^

**FIGURE 1:**
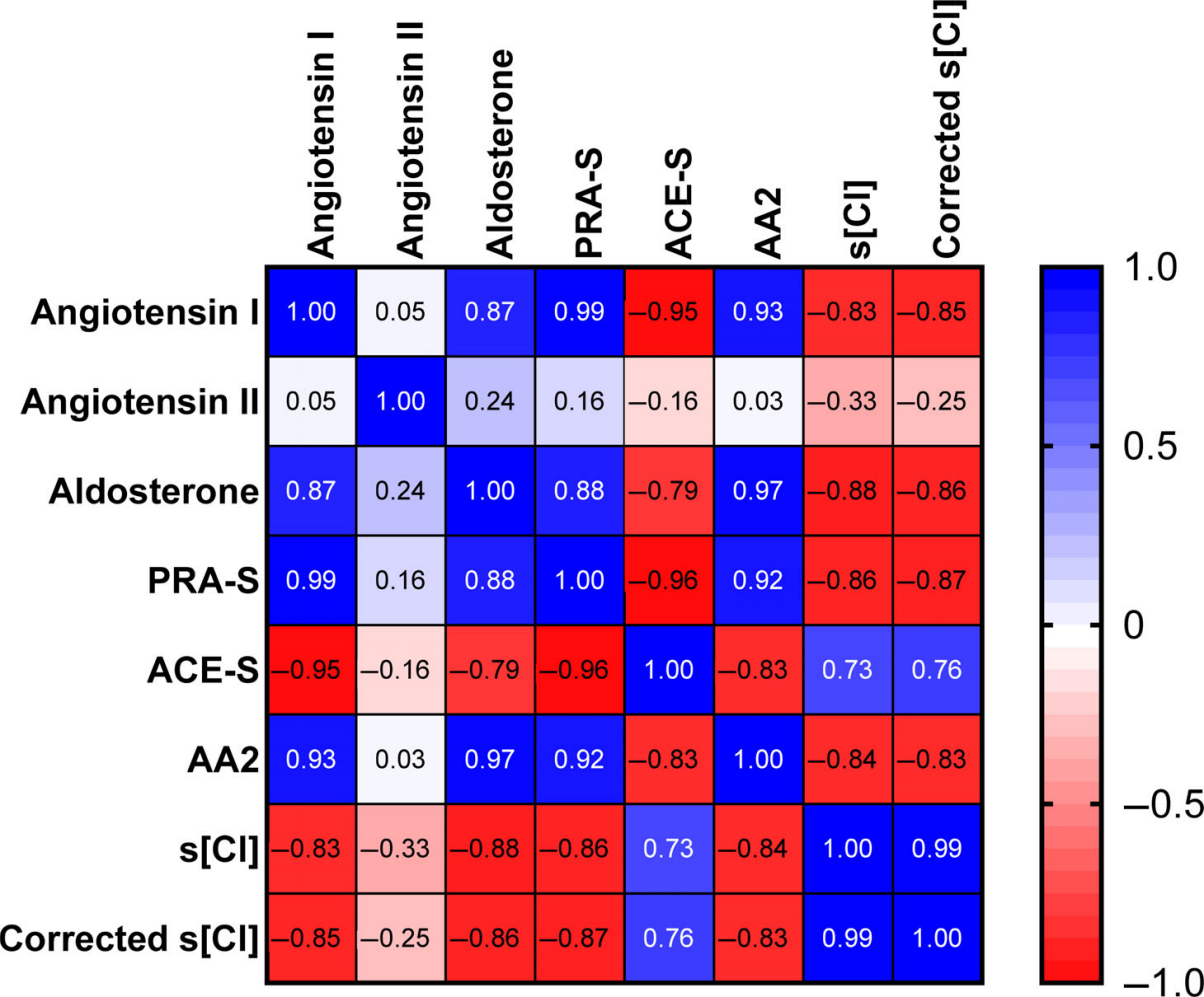
Pearson's correlation heatmap shows the relationships between measured variables on 6 different days during hospitalization for the dog in this report. Positive correlation coefficients are depicted in blue, and negative correlation coefficients are depicted in red. PRA‐S, renin activity surrogate calculated as the sum of angiotensin I and angiotensin II; ACE‐S, angiotensin‐converting enzyme activity surrogate calculated as angiotensin II divided by angiotensin I; AA2, aldosterone to angiotensin II ratio; s[Cl], serum chloride concentration.

**FIGURE 2:**
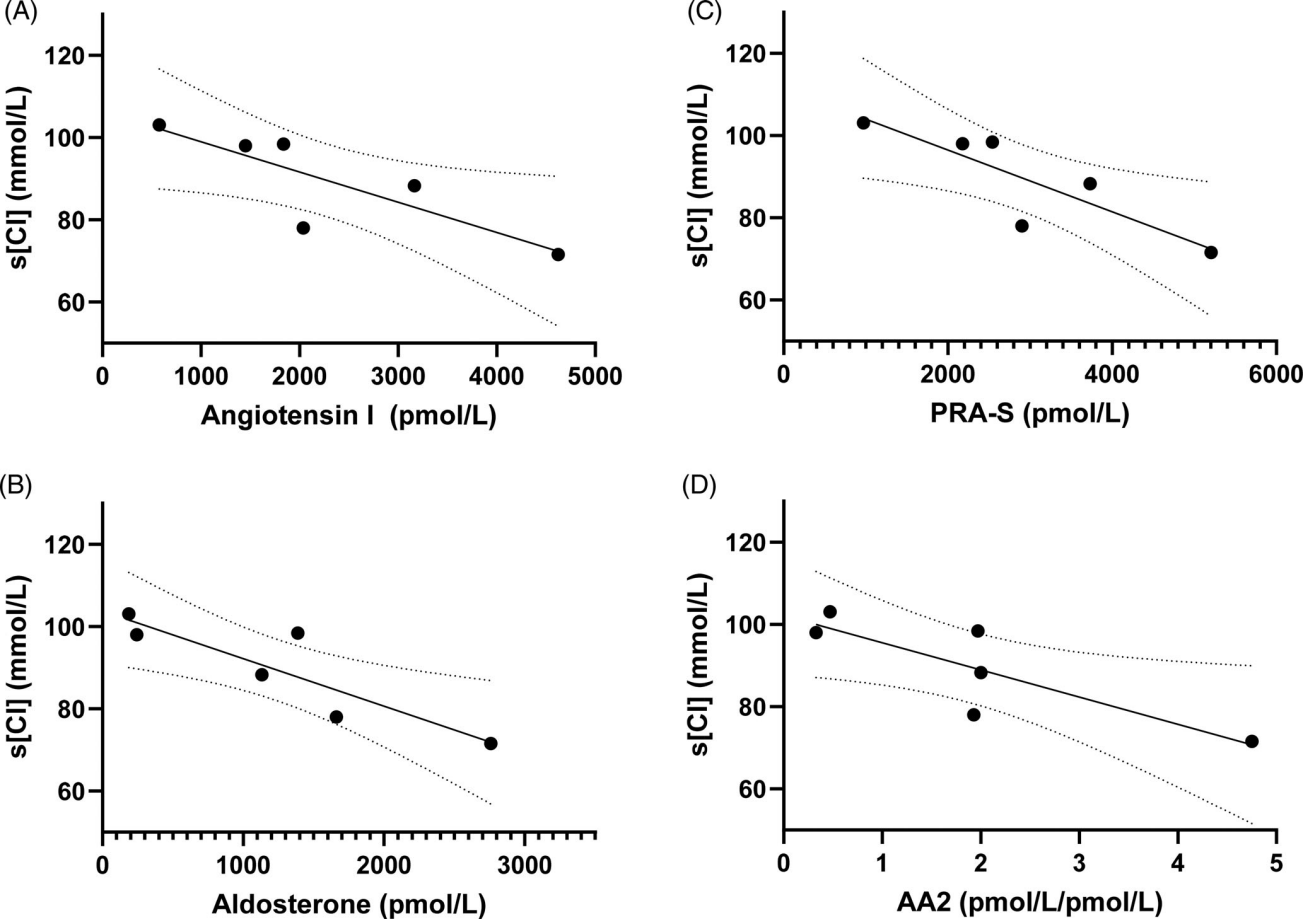
Scatterplots of serum chloride concentrations (s[Cl]) and select renin‐angiotensin‐aldosterone system components fit with a simple linear regression best‐fit line and 95% confidence bands on 6 different days of hospitalization for the dog of this report are shown. Each datapoint represents a different day. (A) Angiotensin I, (B) aldosterone, (C) PRA‐S (renin activity surrogate calculated as the sum of angiotensin I and angiotensin), and (D) AA2 (aldosterone to angiotensin II ratio).

Repeated and paired serum [Cl^−^] and RAAS measurements from this dog at multiple time points were normally distributed (Shapiro‐Wilks test), so Pearson's correlation test was used to explore relationships between variables (Figure 1). Angiotensin I, aldosterone, PRA‐S, and AA2 were strongly negatively correlated with serum [Cl^−^] (Table 2). The strong correlations persisted even when low sodium concentrations were accounted for by mathematically correcting serum [Cl^−^] for a normal sodium concentration (Figure 1). The relationships between serum [Cl^−^] and selected RAAS components are visually shown as scatterplots in Figure 2.

**TABLE 2:** The correlation of renin‐angiotensin‐aldosterone system components to measured serum chloride concentrations on 6 different days of hospitalization for this dog is shown.

Variable	Correlation coefficient (*r*)	95% confidence interval	*P* value
Angiotensin I	−0.8318	−0.9811 to −0.0623	.04
Angiotensin II	−0.3343	−0.9013 to 0.6550	.52
Aldosterone	−0.8835	−0.9872 to −0.2540	.02
PRA‐S	−0.8596	−0.9844 to −0.1590	.03
ACE‐S	0.7287	−0.2028 to 0.9679	.10
AA2	−0.8366	−0.9817 to −0.0781	.04

Abbreviations: AA2, aldosterone to angiotensin II ratio; ACE‐S, angiotensin‐converting enzyme activity surrogate calculated as angiotensin II divided by angiotensin I; PRA‐S, renin activity surrogate calculated as the sum of angiotensin I and angiotensin II.

## 3 DISCUSSION

The prolonged hospitalization resulting from severe CHF in the dog of this report presented a unique opportunity to explore relationships among biomarkers of RAAS activity with serum [Cl^−^] in the absence of RAAS inhibitor medications (such as angiotensin‐converting enzyme inhibitors or spironolactone) and across a wide range of serum [Cl^−^]. Even with a relatively small number of samples, we found a strong inverse relationship between serum [Cl^−^] and indicators of classical RAAS activation (eg, angiotensin I, aldosterone, PRA‐S, AA2). Despite increases at some time points, this dog's mathematically corrected serum [Cl^−^] remained low, suggesting mixed etiology (dilution and depletion) of hypochloremia in this dog.^16^ The relationships with RAAS components were also observed with mathematically corrected [Cl^−^], suggesting that the cause of hypochloremia (dilution vs depletion) might not be an important factor.

Although cause and effect cannot be determined, the findings from this case support a role for enhanced classical RAAS activation with progressive hypochloremia that should be further explored prospectively. There is recent interest in human and veterinary medicine to understand the role of hypochloremia in heart failure because studies in people have demonstrated that hypochloremia is a negative prognostic indicator of this disease.^2^, ^4^, ^17^ Likewise, hypochloremia is a useful marker for heart disease severity and possibly therapeutic response in dogs with CHF.^5^, ^6^ Although hyponatremia has historically been assumed to be prognostically important, recent studies indicate that chloride is more predictive of outcomes than sodium, supporting a future research focus on chloride abnormalities in patients with heart failure.^4^, ^18^, ^19^ Although multiple pathways exist for hypochloremia to develop in heart failure patients, the underlying mechanism for promoting disease progression has not been shown.^3^, ^16^ The inverse relationship between serum [Cl^−^] and maladaptive RAAS components in the dog of this report suggests that RAAS dysregulation might be mechanistically important.

We did not find a relationship between serum [Cl^−^] and angiotensin II. The reason for this is unknown, but angiotensin II production could be regulated by other pathways or hypochloremia might have reduced ACE activity and interrupted the relationship because chloride is required for ACE activation.^20^, ^21^, ^22^ Additionally, ACE utilizes a wide range of substrates, so the effect of hypochloremia on enzyme activation could be complex.^23^ Although it did not reach statistical significance in this small sampling, the relationship of ACE‐S to [Cl^−^] appeared to be direct, supporting chloride's role in enzyme activation.^21^, ^24^ This dog did not receive ACE inhibitor medication, making the interpretation of the results surrounding the RAAS cascade clearer than if medical inhibition was provided. Endogenous ACE activity in refractory CHF dogs is lower compared to controlled C CHF dogs, and refractory CHF dogs are also characterized by more pronounced hypochloremia.^5^ The ACE polymorphism heterozygosity of this Cavalier King Charles Spaniel mostly likely did not alter measured and calculated RAAS components in light of previous work showing unchanged angiotensin II concentrations despite lower ACE activity in polymorphism positive dogs.^15^ Although a greater prevalence of aldosterone breakthrough was found in dogs positive for the ACE polymorphism after treatment with ACE inhibitor therapy, the dog of this report did not receive ACE inhibitors.

Firm conclusions cannot be drawn from a single case report. Still, the repeated, paired sampling and wide range of serum [Cl^−^] in these samples provide pilot information to justify performing a prospective study in dogs with naturally occurring heart failure. In addition to the results obtained from a single dog, other aspects that limit interpretation include incomplete assessment of the RAAS cascade (we did not evaluate alternative RAAS components or direct enzyme activities) and other clinical variables, including numerous medications associated with a hospitalized dog that could have negatively or positively affected any of the RAAS variables assessed in this report. For instance, amlodipine administration, which was started on Day 5 of hospitalization, activates the RAAS,^25^ and this timing coincides with when the dog in this report exhibited the highest concentrations of angiotensin I, aldosterone, and PRA‐S. Overall, however, medications were relatively stable during this period, the dog did not receive ACE inhibitors, and a wide range of serum [Cl^−^] was found in these samples.

Repeated evaluation of blood samples from this dog hospitalized for treatment of CHF over 12 days showed an inverse relationship between serum [Cl^−^] and angiotensin I, aldosterone, renin activity, and adrenal responsiveness to angiotensin II. These findings provide insight into the possible mechanism by which hypochloremia is associated with advanced heart disease and poor outcomes in CHF and support efforts to address hypochloremia in this patient population.

## CONFLICT OF INTEREST DECLARATION

Dr Shoemaker is employed by the University of Kentucky Mass Spectrometry and Proteomics Core Facility. No other authors declare a conflict of interest.

## OFF‐LABEL ANTIMICROBIAL DECLARATION

Authors declare no off‐label use of antimicrobials.

## INSTITUTIONAL ANIMAL CARE AND USE COMMITTEE (IACUC) OR OTHER APPROVAL DECLARATION

Authors declare no IACUC or other approval was needed.

## HUMAN ETHICS APPROVAL DECLARATION

Authors declare human ethics approval was not needed for this study.
